# ^1^H-MRS Measured Ectopic Fat in Liver and Muscle in Danish Lean and Obese Children and Adolescents

**DOI:** 10.1371/journal.pone.0135018

**Published:** 2015-08-07

**Authors:** Cilius Esmann Fonvig, Elizaveta Chabanova, Ehm Astrid Andersson, Johanne Dam Ohrt, Oluf Pedersen, Torben Hansen, Henrik S. Thomsen, Jens-Christian Holm

**Affiliations:** 1 The Children’s Obesity Clinic, Department of Pediatrics, Copenhagen University Hospital Holbæk, Holbæk, Denmark; 2 The Novo Nordisk Foundation Center for Basic Metabolic Research, Section of Metabolic Genetics, Faculty of Medical and Health Sciences, University of Copenhagen, Copenhagen Ø, Denmark; 3 Department of Diagnostic Radiology, Copenhagen University Hospital Herlev, Herlev, Denmark; 4 University of Southern Denmark, Faculty of Health Sciences, Odense, Denmark; 5 University of Copenhagen, Faculty of Medical and Health Sciences, Copenhagen N, Denmark; NIH / NIDDK, UNITED STATES

## Abstract

**Objectives:**

This cross sectional study aims to investigate the associations between ectopic lipid accumulation in liver and skeletal muscle and biochemical measures, estimates of insulin resistance, anthropometry, and blood pressure in lean and overweight/obese children.

**Methods:**

Fasting plasma glucose, serum lipids, serum insulin, and expressions of insulin resistance, anthropometry, blood pressure, and magnetic resonance spectroscopy of liver and muscle fat were obtained in 327 Danish children and adolescents aged 8–18 years.

**Results:**

In 287 overweight/obese children, the prevalences of hepatic and muscular steatosis were 31% and 68%, respectively, whereas the prevalences in 40 lean children were 3% and 10%, respectively. A multiple regression analysis adjusted for age, sex, body mass index z-score (BMI SDS), and pubertal development showed that the OR of exhibiting dyslipidemia was 4.2 (95%CI: [1.8; 10.2], *p* = 0.0009) when hepatic steatosis was present. Comparing the simultaneous presence of hepatic and muscular steatosis with no presence of steatosis, the OR of exhibiting dyslipidemia was 5.8 (95%CI: [2.0; 18.6], *p* = 0.002). No significant associations between muscle fat and dyslipidemia, impaired fasting glucose, or blood pressure were observed.

Liver and muscle fat, adjusted for age, sex, BMI SDS, and pubertal development, associated to BMI SDS and glycosylated hemoglobin, while only liver fat associated to visceral and subcutaneous adipose tissue and intramyocellular lipid associated inversely to high density lipoprotein cholesterol.

**Conclusion:**

Hepatic steatosis is associated with dyslipidemia and liver and muscle fat depositions are linked to obesity-related metabolic dysfunctions, especially glycosylated hemoglobin, in children and adolescents, which suggest an increased cardiovascular disease risk.

## Introduction

The liver and skeletal muscles are main ectopic sites of excess fat storage in obese subjects, particularly when the capacity of energy storage in the adipose tissue is exceeded [1]. Fat deposition in non-adipose tissues (i.e. liver and skeletal muscle) may act as an inflammatory mediator [1] and is associated with systemic insulin resistance (IR) [2,3]. Furthermore, the ectopic fat deposition in liver and skeletal muscle tissue is associated with the metabolic syndrome and an increased risk of cardiovascular disease [1,2,4,5].

Dyslipidemia and fat deposition in liver and skeletal muscle may be present already in childhood [6–8] with reported prevalence rates of both hepatic and muscular steatosis among obese children of up to around 80% [7–9]. The non-invasive and non-ionizing proton magnetic resonance spectroscopy (MRS) has a high accuracy in detecting and quantifying hepatic steatosis [10,11], and due to advances in muscle lipid imaging, MRS has facilitated the differentiation of lipid deposits of skeletal muscle in intramyocellular lipid (IMCL) and extramyocellular lipid (EMCL) [12]. Especially, the accumulation of IMCL has been given attention in the literature, since it has been demonstrated to be inversely associated with insulin sensitivity in adults and adolescents [3,4,13].

The objective of the present cross sectional study was to elucidate the differences of liver and skeletal muscle lipid accumulation between Danish lean and overweight/obese children and adolescents and, furthermore, to investigate the associations between liver and muscle lipid accumulation and fasting plasma glucose, serum lipids, serum insulin, the homeostatic model assessment of insulin resistance (HOMA-IR), glycosylated hemoglobin (HbA1c), anthropometrics, and blood pressure (BP).

## Methods

### Study population

From August 2009 to August 2014, 302 overweight children and adolescents were enrolled in the chronic care multidisciplinary intervention program at The Children’s Obesity Clinic, Department of Pediatrics, Copenhagen University Hospital Holbæk, Denmark [14] who concomitantly had a magnetic resonance spectroscopy (MRS) assessment of ectopic lipid accumulation in liver and skeletal muscle within 60 days of anthropometric measures in the clinic, and estimates of fasting plasma glucose and serum lipids and insulin. The inclusion criteria for the study group in the present study were i) 8–18 years of age, ii) enrollment in childhood obesity treatment, and iii) a body mass index (BMI) standard deviation score (SDS) above 1.28, which corresponds to the 90^th^ percentile according to Danish age- and sex-adjusted references [15]. The overweight group (the case group) was assessed at treatment enrollment. From April 2012 through August 2014, 63 age- and sex matched controls were recruited from schools in the same geographical regions (Capital Region and Region Zealand, Denmark). The inclusion criteria for the control group were i) 8–18 years of age and ii) a BMI SDS between -1.28 and 1.28 corresponding to the 10^th^ and 90^th^ BMI percentile, respectively [15].

The exclusion criteria for both groups were i) a body weight above 135 kg, which was the maximum capacity of the MR scanner, ii) inability to remain quiet in the MR machine during the 45 minutes scan time, iii) a fasting plasma glucose concentration of 7.0 mmol/L or above, or iv) an alcohol consumption of more than 140 g/week.

### Anthropometry

Body weight was measured on a Tanita digital medical scale (WB-100 MA; Tanita Corp., Tokyo, Japan) to the nearest 0.1 kg. Height was measured by stadiometer to the nearest 1 mm. Weight and height were measured without shoes in underwear or light indoor clothing. BMI was calculated as weight divided by height squared (kg/m^2^). The BMI SDS was calculated by the LMS method by converting BMI into a normal distribution by sex and age using the median coefficient of variation and a measure of the skewness [16], based on the Box-Cox power plot based on Danish BMI charts [15]. Waist circumference was measured to the nearest 1 mm at the level of the umbilicus with a non-elastic tape measure, standing with arms down after a gentle expiration.

### Pubertal development

The pubertal stage in the overweight/obese children was determined by a trained pediatrician using the classification of Tanner [17]. In boys, the development stages of pubic hair and genitals were determined, and in girls, the development stages of breast and pubic hair were determined. The pubertal development in the lean control group was assessed by a questionnaire with illustrations and a supplementary text of the five Tanner stages in each category [17].

### MR spectroscopy and imaging

MR measurements were performed on a 3.0 T MR imaging system (Achieva, Philips Medical Systems, Best, The Netherlands) using a SENSE cardiac coil. The participants were examined in the supine position. Liver fat content (LFC), muscle fat content (MFC), and the muscular fragments of IMCL and EMCL were simultaneously measured by MRS. Muscle fat fractions were all measured in the psoas muscle. Volumes of visceral adipose tissue (VAT) and subcutaneous adipose tissue (SAT) were measured by magnetic resonance imaging (MRI), where data were assessed from the level of the third lumbar vertebra with a transverse slice of 10 mm thickness. The spectroscopy volume (11 mm x 11 mm x 11mm) in the liver was positioned in the right lobe. The position of the volume was individually determined for each subject in order to avoid the vascular structures. The acquired spectra were fitted to obtain peak areas using standard post processing protocol for fitting metabolite peak areas available at the Achieva 3.0 T MR imaging system. Mathematical and statistical calculations were performed using the MATLAB software. The volumes of visceral and subcutaneous fat at L3 were measured in cm^3^ using “segmentation tool” in “volume analysis” on the Philips ViewForum workstation. Data post processing was performed by a senior experienced MR physicist. The details of the applied methodology of MR spectroscopy and imaging in the present study have previously been described [7,8,18].

Hepatic steatosis was defined as an LFC >5% [19], and muscular steatosis was defined as an MFC >5% [8].

### Blood sampling

Blood samples were drawn intravenously from an antecubital vein between 7 a.m. and 9 a.m. after an overnight fast. If required, an anesthetic cream was applied 60 minutes before venipuncture. The biochemical analyses of serum concentrations of triglycerides, total cholesterol, and high density lipoprotein (HDL) cholesterol, and concentrations of plasma glucose were performed immediately after sampling on a Dimension Vista 1500 analyzer (Siemens, Munich, Germany). Plasma glucose samples were collected in fluoride containing tubes. The serum samples of triglycerides and cholesterol fractions and the plasma glucose samples were stored at room temperature for less than 30 minutes after sampling before being centrifuged at four degrees Celsius. Serum insulin was collected in a tube containing serum separating gel. The biochemical analyses of insulin were performed on a Cobas 6000 analyzer (F. Hoffmann-La Roche Ltd, Basel, Switzerland) and stored at room temperature for 30–60 minutes after sampling before being centrifuged at four degrees Celsius. Whole blood HbA1c was analyzed on a Tosoh high-performance liquid chromatography G8 analyzer (Tosoh Corporation, Tokyo, Japan). Low density lipoprotein concentration was calculated as: Total cholesterol–(triglycerides x 0.45) + HDL cholesterol. Non-HDL was calculated as: Total cholesterol–HDL cholesterol. HOMA-IR was calculated as: Insulin (pmol/l) x glucose (mmol/l) / (22 x 6.945).

Dyslipidemia was defined as a total cholesterol concentration >5.18 mmol/l (200 mg/dl), HDL cholesterol concentration <0.91 mmol/l (35 mg/dl), low density lipoprotein cholesterol concentration >3.37 mmol/l (130 mg/dl), or triglyceride concentration >1.69 mmol/l (150 mg/dl) according to reference guidelines [20].

Impaired fasting plasma (IFG) was defined as a fasting plasma glucose concentration in the range 5.6–6.9 mmol/l according to the 2011 International Diabetes Federation and International Society for Pediatric and Adolescent Diabetes guidelines for diabetes in childhood and adolescence [21].

### Blood pressure

BP was measured three times after a rest of minimum 5 minutes in the supine position with the standardized oscillometric device Omron 705IT (OMRON Healthcare, Japan). Mean of the last two out of three BP measurements was reported and calculated into SDS according to an American reference population based on sex, age, and height [22]. Hypertension was defined as a systolic or diastolic BP ≥ 95^th^ percentile (≥ 1.64 BP SDS) according to the reference population [22].

### Statistical analysis

Statistical analyses were performed using “R” statistical software version 3.1.2 (http://www.r-project.org). In Table 1, differences in continuous variables between groups were analyzed by Wilcoxon signed rank test. All two by two comparisons were analyzed by Fisher’s exact test. To investigate associations, multiple linear regression models where used in the Tables 2–5 and logistic regression models of the binomial family were used in the assessment of odds ratios (ORs). *P*-values were not adjusted for multiple hypothesis testing. The level of significance was set at *p* <0.05 and power calculations were performed with a type I error rate of 0.05. This study has a 95% statistical power to detect a 0.5 percentage-point difference in liver fat content between two lean and two overweight/obese children and adolescents per age represented, when assuming a standard deviation of 2.5 (the expected in lean children would be less than 2 [23]). Likewise, this study has a 95% statistical power to detect a 0.5 percentage-points difference in muscle fat content between two lean and two overweight/obese children and adolescents per age represented, when assuming a standard deviation of 2.0 (the expected in lean children would be less than 1 [23]).

**Table 1:** Baseline characteristics of 287 overweight/obese (cases) and 40 lean (controls) children and adolescents.

	Cases Girls ♀	Controls Girls ♀		Cases Boys ♂	Controls Boys ♂		Cases ♀ *vs* ♂	Controls ♀ *vs* ♂
N	165	19		122	21		287	40
Age, *years*	13.3 (11.6–15.0)	14.8 (12.1–16.4)	*0*.*13*	12.7 (11.5–14.3)	14.2 (11.9–16.1)	*0*.*08*	*0*.*07*	*0*.*77*
BMI SDS	2.73 (2.38–3.04)	-0.06 (-0.38–0.41)	***<0*.*0001***	3.07 (2.73–3.48)	0.39 (-0.21–0.54)	***<0*.*0001***	***<0*.*0001***	*0*.*54*
VAT, *cm^3^*	73 (54–99)	13 (12–22)	***<0*.*0001***	84 (60–117)	13 (11–21)	***<0*.*0001***	***0*.*04***	*1*.*00*
SAT, *cm^3^*	298 (237–383)	56 (45–84)	***<0*.*0001***	289 (236–385)	37 (30–53)	***<0*.*0001***	*0*.*71*	***0*.*02***
LFC, %	3.0 (1.5–4.5)	1.5 (1.5–1.7)	***0*.*002***	3.5 (2.0–10.7)	1.5 (1.5–1.5)	***<0*.*0001***	***0*.*0005***	*0*.*46*
Hepatic steatosis, *fraction*	23% (38/165)	5% (1/19)	*0*.*08*	41% (50/122)	0% (0/21)	***<0*.*0001***	***0*.*001***	*0*.*48*
MFC, %	7.3 (4.2–10.6)	2.6 (0.7–4.3)	***<0*.*0001***	6.9 (4.6–11.7)	1.0 (1.0–2.7)	***<0*.*0001***	*0*.*72*	*0*.*51*
Muscle steatosis, *fraction*	68% (113/165)	11% (2/19)	***<0*.*0001***	68% (83/122)	10% (2/21)	***<0*.*0001***	*1*.*00*	*1*.*00*
IMCL, %	1.6 (1.0–2.8)	0.5 (0.3–1.7)	***0*.*0003***	1.9 (1.1–3.0)	0.5 (0.4–0.8)	***<0*.*0001***	*0*.*14*	*0*.*94*
EMCL, %	5.6 (2.6–8.1)	1.0 (0.3–3.0)	***<0*.*0001***	5.1 (3.0–8.4)	0.5 (0.5–1.4)	***<0*.*0001***	*0*.*91*	*0*.*44*
Triglyceride, *mmol/l*	1.1 (0.8–1.4)	0.7 (0.5–0.9)	***0*.*0002***	0.9 (0.7–1.6)	0.5 (0.4–0.7)	***<0*.*0001***	*0*.*08*	*0*.*06*
HDL cholesterol, *mmol/l*	1.1 (1.0–1.3)	1.7 (1.3–1.8)	***<0*.*0001***	1.2 (1.0–1.4)	1.8 (1.4–2.1)	***<0*.*0001***	*0*.*10*	*0*.*22*
LDL cholesterol, *mmol/l*	2.3 (2.0–2.9)	2.3 (2.1–2.8)	*0*.*82*	2.6 (2.1–3.0)	1.9 (1.6–2.5)	***0*.*005***	*0*.*18*	*0*.*07*
Non-HDL cholesterol, *mmol/l*	2.9 (2.5–3.5)	2.6 (2.4–3.1)	*0*.*10*	3.0 (2.5–3.8)	2.2 (1.8–2.8)	***0*.*0003***	*0*.*56*	***0*.*03***
Plasma glucose, *mmol/l*	5.0 (4.7–5.3)	5.0 (4.9–5.4)	*0*.*28*	5.2 (4.9–5.5)	5.1 (4.6–5.4)	*0*.*34*	***0*.*001***	*0*.*67*
Serum insulin, *pmol/l*	89 (61–131)	77 (48–92)	*0*.*05*	93 (67–131)	46 (30–63)	***<0*.*0001***	*0*.*69*	***0*.*004***
HbA1c, *mmol/l*	34 (32–37)	36 (35–37)	***0*.*03***	34 (32–37)	34 (32–36)	*0*.*45*	*0*.*73*	***0*.*02***
HOMA-IR	2.87 (1.84–4.27)	2.31 (1.62–2.82)	*0*.*09*	3.08 (2.19–4.39)	1.61 (0.94–2.04)	***<0*.*0001***	*0*.*43*	***0*.*007***
sysBP SDS	2.32 (1.48–2.89)	1.52 (0.94–1.89)	***0*.*001***	1.99 (1.05–3.03)	2.19 (1.28–3.19)	*0*.*44*	*0*.*27*	***0*.*047***
diaBP SDS	1.03 (0.53–1.43)	0.33 (0.22–0.81)	***0*.*001***	0.65 (0.22–1.09)	0.65 (0.03–0.81)	*0*.*12*	***0*.*0004***	*0*.*98*
Tanner stage	4 (2–4)	4 (2–5)	*0*.*79*	2 (1–3)	4 (1–4)	*0*.*08*	***<0*.*0001***	*0*.*65*

Data are presented as medians (interquartile range) due to a non-normal distribution. BMI, body mass index; diaBP, diastolic blood pressure; EMCL, extramyocellular lipid content; HbA1c, glycosylated hemoglobin; HDL, high density lipoprotein; HOMA-IR, homeostatic model assessment of insulin resistance; IMCL, intramyocellular lipid content; LDL, low density lipoprotein; LFC, liver fat content; MFC, muscle fat content; SAT, subcutaneous adipose tissue volume; SDS, standard deviation score; sysBP, systolic blood pressure; VAT, visceral adipose tissue volume.

**Table 2:** Multiple linear regression showing the relationship between liver fat content and markers of fat distribution, insulin resistance, and cardiovascular risk in the 287 overweight/obese children and adolescents.

	Model A			Model B			Model C		
	β(SE)	R^2^	*p*	β(SE)	R^2^	*p*	β(SE)	R^2^	*p*
**LFC as the dependent variable**									
Basic model		0.04			0.11			0.12	
Muscle fat content, *%*	0.23 (0.12)	0.06	*0*.*06*	0.16 (0.12)	0.12	*0*.*18*	0.16 (0.12)	0.12	*0*.*18*
BMI SDS	4.76 (1.36)	0.11	***0*.*0006***	4.76 (1.36)	0.11	***0*.*0006***	4.44 (1.38)	0.12	***0*.*02***
VAT, *cm^3^*	0.09 (0.02)	0.21	***<0*.*0001***	0.08 (0.02)	0.21	***<0*.*0001***	0.07 (0.02)	0.22	***<0*.*0001***
SAT, *cm^3^*	0.03 (0.01)	0.13	***0*.*0002***	0.02 (0.01)	0.13	***0*.*07***	0.02 (0.01)	0.15	***0*.*048***
**LFC as the independent variable**									
Triglyceride, *mmol/l*	0.01 (0.01)	0.08	*0*.*06*	0.01 (0.01)	0.08	*0*.*08*	0.01 (0.01)	0.08	*0*.*09*
HDL cholesterol, *mmol/l*	-0.00 (0.00)	0.09	***0*.*04***	-0.00 (0.00)	0.09	*0*.*07*	-0.00 (0.00)	0.11	*0*.*10*
Plasma glucose, *mmol/*	0.00 (0.00)	0.07	*0*.*22*	0.00 (0.00)	0.08	*0*.*41*	0.00 (0.00)	0.08	*0*.*43*
Serum insulin, *pmol/l*	2.06 (0.79)	0.10	***0*.*01***	1.17 (0.78)	0.19	*0*.*14*	1.20 (0.79)	0.19	*0*.*13*
HbA1c, *mmol/l*	0.10 (0.03)	0.09	***0*.*0008***	0.09 (0.03)	0.09	***0*.*004***	0.09 (0.03)	0.11	***0*.*007***
HOMA-IR	0.07 (0.03)	0.10	***0*.*01***	0.04 (0.03)	0.20	*0*.*13*	0.04 (0.03)	0.20	*0*.*13*
sysBP SDS	0.00 (0.01)	0.24	*0*.*59*	-0.01 (0.01)	0.33	*0*.*56*	0.01 (0.01)	0.33	*0*.*67*
diaBP SDS	0.00 (0.01)	0.17	*0*.*92*	-0.01 (0.01)	0.25	*0*.*36*	-0.01 (0.01)	0.25	*0*.*35*

Estimates (β), standard errors (SE), and correlation coefficients (R^2^) of the relationship between liver fat content and markers of fat distribution, insulin resistance, and cardiovascular risk. The “basic model” compromises age, sex, and pubertal development. Model A includes the basic model, liver fat content, and the variable mentioned. Model B is model A adjusted for BMI SDS. Model C is model B adjusted for muscle fat content. BMI, body mass index; diaBP, diastolic blood pressure; HbA1c, glycosylated hemoglobin; HDL, high density lipoprotein; HOMA-IR, homeostasis model assessment of insulin resistance; LFC, liver fat content; SAT, subcutaneous adipose tissue volume; SDS, standard deviation score; sysBP, systolic blood pressure; VAT, visceral adipose tissue volume.

**Table 3:** Multiple linear regression showing the relationship between muscle fat content and markers of fat distribution, insulin resistance, and cardiovascular risk in the 287 overweight/obese children and adolescents.

	Model A			Model B			Model C		
	β(SE)	R^2^	*p*	β(SE)	R^2^	*p*	β(SE)	R^2^	*p*
**MFC as the dependent variable**									
Basic model		0.03			0.06			0.07	
Liver fat content, *%*	0.10 (0.05)	0.05	*0*.*06*	0.07 (0.05)	0.07	*0*.*18*	0.07 (0.05)	0.07	*0*.*18*
BMI SDS	1.96 (0.92)	0.06	***0*.*03***	1.96 (0.92)	0.06	***0*.*03***	1.61 (0.95)	0.07	*0*.*09*
VAT, *cm^3^*	0.03 (0.01)	0.07	***0*.*02***	0.02 (0.01)	0.07	*0*.*11*	0.02 (0.01)	0.08	*0*.*21*
SAT, *cm^3^*	0.00 (0.01)	0.03	*0*.*46*	-0.01 (0.01)	0.07	*0*.*17*	-0.01 (0.01)	0.08	*0*.*11*
**MFC as the independent variable**									
Triglyceride, *mmol/l*	0.01 (0.01)	0.06	*0*.*38*	0.01 (0.01)	0.06	*0*.*44*	0.01 (0.01)	0.08	*0*.*55*
HDL cholesterol, *mmol/l*	-0.01 (0.00)	0.09	***0*.*049***	-0.01 (0.00)	0.09	*0*.*08*	-0.01 (0.00)	0.11	*0*.*11*
Plasma glucose, *mmol/*	0.00 (0.01)	0.06	*0*.*50*	0.00 (0.01)	0.08	*0*.*68*	0.00 (0.01)	0.08	*0*.*75*
Serum insulin, *pmol/l*	0.67 (1.24)	0.06	*0*.*59*	-0.27 (1.18)	0.18	*0*.*82*	-0.47 (1.18)	0.19	*0*.*69*
HbA1c, *mmol/l*	0.11 (0.04)	0.05	***0*.*02***	0.10 (0.05)	0.07	***0*.*04***	0.08 (0.04)	0.11	*0*.*07*
HOMA-IR	0.03 (0.04)	0.06	*0*.*50*	0.00 (0.04)	0.18	*0*.*92*	-0.01 (0.04)	0.20	*0*.*79*
sysBP SDS	-0.01 (0.01)	0.24	*0*.*54*	-0.02 (0.01)	0.33	*0*.*17*	-0.02 (0.01)	0.33	*0*.*19*
diaBP SDS	0.01 (0.01)	0.17	*0*.*47*	0.00 (0.01)	0.24	*0*.*88*	0.00 (0.01)	0.25	*0*.*80*

Estimates (β), standard errors (SE), and correlation coefficients (R^2^) of the relationship between muscle fat content and markers of fat distribution, insulin resistance, and cardiovascular risk. The “basic model” compromises age, sex, and pubertal development. Model A includes the basic model, muscle fat content, and the variable mentioned. Model B is model A adjusted for BMI SDS. Model C is model B adjusted for liver fat content. BMI, body mass index; diaBP, diastolic blood pressure; HbA1c, glycosylated hemoglobin; HDL, high density lipoprotein; HOMA-IR, homeostasis model assessment of insulin resistance; MFC, muscle fat content; SAT, subcutaneous adipose tissue volume; SDS, standard deviation score; sysBP, systolic blood pressure; VAT, visceral adipose tissue volume.

**Table 4:** Multiple linear regression showing the relationship between intramyocellular lipid content and markers of fat distribution, insulin resistance, and cardiovascular risk in the 287 overweight/obese children and adolescents.

	Model A			Model B			Model C		
	β(SE)	R^2^	*p*	β(SE)	R^2^	*p*	β(SE)	R^2^	*p*
**IMCL as the dependent variable**									
Basic model		0.03			0.07			0.07	
Liver fat content, *%*	0.01 (0.02)	0.03	*0*.*72*	-0.01 (0.02)	0.07	*0*.*71*	-0.01 (0.02)	0.07	*0*.*71*
BMI SDS	0.87 (0.34)	0.07	***0*.*01***	0.87 (0.34)	0.07	***0*.*01***	0.90 (0.36)	0.07	***0*.*01***
VAT, *cm^3^*	0.01 (0.00)	0.04	*0*.*09*	0.00 (0.00)	0.07	*0*.*53*	0.00 (0.00)	0.07	*0*.*42*
SAT, *cm^3^*	0.00 (0.00)	0.03	*0*.*25*	0.00 (0.00)	0.08	*0*.*24*	0.00 (0.00)	0.08	*0*.*26*
**IMCL as the independent variable**									
Triglyceride, *mmol/l*	-0.01 (0.02)	0.04	*0*.*79*	-0.01 (0.02)	0.05	*0*.*67*	-0.01 (0.02)	0.07	*0*.*71*
HDL cholesterol, *mmol/l*	-0.02 (0.01)	0.10	***0*.*03***	-0.02 (0.01)	0.10	***0*.*04***	-0.02 (0.01)	0.12	***0*.*04***
Plasma glucose, *mmol/*	-0.02 (0.02)	0.07	*0*.*30*	-0.02 (0.02)	0.09	*0*.*17*	-0.02 (0.02)	0.09	*0*.*18*
Serum insulin, *pmol/l*	2.41 (3.50)	0.06	*0*.*49*	-0.67 (3.37)	0.17	*0*.*84*	-0.49 (3.36)	0.18	*0*.*88*
HbA1c, *mmol/l*	0.37 (0.13)	0.07	***0*.*004***	0.34 (0.13)	0.08	***0*.*009***	0.34 (0.12)	0.14	***0*.*006***
HOMA-IR	0.08 (0.12)	0.06	*0*.*51*	-0.03 (0.12)	0.17	*0*.*81*	-0.02 (0.12)	0.19	*0*.*85*
sysBP SDS	-0.03 (0.04)	0.24	*0*.*49*	-0.06 (0.04)	0.33	*0*.*12*	-0.06 (0.04)	0.33	*0*.*12*
diaBP SDS	0.05 (0.03)	0.19	*0*.*07*	0.03 (0.03)	0.23	*0*.*20*	0.03 (0.03)	0.23	*0*.*22*

Estimates (β), standard errors (SE), and correlation coefficients (R^2^) of the relationship between IMCL and markers of fat distribution, insulin resistance, and cardiovascular risk. The “basic model” compromises age, sex, and pubertal development. Model A includes the basic model, intramyocellular lipid content, and the variable mentioned. Model B is model A adjusted for BMI SDS. Model C is model B adjusted for liver fat content. BMI, body mass index; diaBP, diastolic blood pressure; HbA1c, glycosylated hemoglobin; HDL, high density lipoprotein; HOMA-IR, homeostasis model assessment of insulin resistance; IMCL, intramyocellular lipid content; SAT, subcutaneous adipose tissue volume; SDS, standard deviation score; sysBP, systolic blood pressure; VAT, visceral adipose tissue volume.

**Table 5:** Multiple linear regression showing the relationship between extramyocellular lipid content and markers of fat distribution, insulin resistance, and cardiovascular risk in the 287 overweight/obese children and adolescents.

	Model A			Model B			Model C		
	β(SE)	R^2^	*p*	β(SE)	R^2^	*p*	β(SE)	R^2^	*p*
**EMCL as the dependent variable**									
Basic model		0.03			0.05			0.05	
Liver fat content, *%*	0.08 (0.04)	0.05	*0*.*07*	0.07 (0.05)	0.06	*0*.*16*	0.07 (0.05)	0.06	*0*.*16*
BMI SDS	1.28 (0.79)	0.05	*0*.*11*	1.28 (0.79)	0.05	*0*.*11*	0.95 (0.83)	0.06	*0*.*25*
VAT, *cm^3^*	0.02 (0.01)	0.06	***0*.*03***	0.02 (0.01)	0.06	*0*.*11*	0.01 (0.01)	0.07	*0*.*24*
SAT, *cm^3^*	0.00 (0.00)	0.03	*0*.*52*	-0.01 (0.01)	0.05	*0*.*37*	-0.01 (0.01)	0.07	*0*.*26*
**EMCL as the independent variable**									
Triglyceride, *mmol/l*	0.02 (0.01)	0.06	*0*.*15*	0.01 (0.01)	0.06	*0*.*17*	0.01 (0.01)	0.08	*0*.*24*
HDL cholesterol, *mmol/l*	-0.01 (0.00)	0.08	*0*.*15*	-0.01 (0.00)	0.09	*0*.*20*	-0.00 (0.00)	0.10	*0*.*28*
Plasma glucose, *mmol/*	0.01 (0.01)	0.08	*0*.*22*	0.01 (0.01)	0.08	*0*.*29*	0.01 (0.01)	0.09	*0*.*34*
Serum insulin, *pmol/l*	0.55 (1.51)	0.06	*0*.*72*	-0.29 (1.44)	0.17	*0*.*84*	-0.55 (1.44)	0.18	*0*.*70*
HbA1c, *mmol/l*	0.10 (0.05)	0.04	*0*.*08*	0.09 (0.05)	0.05	*0*.*12*	0.07 (0.05)	0.11	*0*.*22*
HOMA-IR	0.03 (0.05)	0.06	*0*.*58*	0.00 (0.05)	0.17	*0*.*99*	-0.01 (0.05)	0.19	*0*.*85*
sysBP SDS	0.00 (0.02)	0.24	*0*.*81*	-0.01 (0.02)	0.32	*0*.*46*	-0.01 (0.02)	0.32	*0*.*50*
diaBP SDS	0.00 (0.01)	0.17	*0*.*84*	0.00 (0.01)	0.22	*0*.*85*	0.00 (0.01)	0.23	*0*.*92*

Estimates (β), standard errors (SE), and correlation coefficients (R^2^) of the relationship between EMCL and markers of fat distribution, insulin resistance, and cardiovascular risk. The “basic model” compromises age, sex, and pubertal development. Model A includes the basic model, extramyocellular lipid content, and the variable mentioned. Model B is model A adjusted for BMI SDS. Model C is model B adjusted for liver fat content. BMI, body mass index; diaBP, diastolic blood pressure; EMCL, extramyocellular lipid content; HbA1c, glycosylated hemoglobin; HDL, high density lipoprotein; HOMA-IR, homeostasis model assessment of insulin resistance; SAT, subcutaneous adipose tissue volume; SDS, standard deviation score; sysBP, systolic blood pressure; VAT, visceral adipose tissue volume.

### Ethical aspects

Informed written consent was obtained from the parents of study participants younger than 18 years and from study participants 18 years of age and older. Additionally, informed assent was provided by all study participants younger than 18 years of age. The study was approved by the Ethics Committee of Region Zealand, Denmark (ID: SJ-104) and the Danish Data Protection Agency (ID: REG-06-2014), and is registered at ClinicalTrials.gov (ID: NCT00928473).

## Results

In total, 287 overweight and obese children and adolescents fulfilled the inclusion criteria. Eight patients were excluded from the study because they had a body weight >135 kg and seven patients were excluded because they exhibited a fasting plasma glucose concentration of 7.0 mmol/L or above. None of the study participants were excluded due to inability to stay quiet during the scan time or an alcohol consumption of more than 140 g/week. The 287 overweight/obese children and adolescents had a median BMI SDS of 2.89 (range 1.32–4.71) and a median age of 12.9 years (8.2–18.8). Of the 63 schoolchildren enrolled, 23 were excluded due to a BMI SDS >1.28, leaving 40 controls with a median BMI SDS of 0.06 (-1.23–1.18) and a median age of 14.2 years (8.7–18.9) for the study. MRS, MRI, and concomitant biochemical and anthropometric measures were performed on all study participants of whom the characteristics are shown in Table 1.

As expected due to study design, the cases were different from the controls in regards to BMI SDS, fat distribution, and fasting circulating levels of triglycerides and HDL cholesterol (Table 1). In the group of boys, cases were different from controls in regards to insulin, HOMA-IR, LDL, and non-HDL cholesterol, whereas in girls, differences were observed in regards to HbA1c and BP (Table 1), where the female controls had a higher HbA1c than male controls and female cases. Among the overweight/obese children and adolescents compared to the lean, there were higher prevalences of dyslipidemia (41% vs. 15%, *p* = 0.001), while prevalences of IFG (10% vs. 8%, *p* = 0.78) and hypertension (68% vs. 53%, *p* = 0.07) did not differ significantly.

Overweight/obese and lean subjects were comparable in aspects of sex distribution (*p* = 0.24), whereas the lean group was older (*p* = 0.02) and more advanced in pubertal development (*p* = 0.003).

Of the 287 cases, 22 were not of Western European descent. Ethnicity did not significantly affect the fat content in liver or muscle (data not shown). The time between the MR scan and the anthropometric measures was a median of 15 days (range: 0–48), and the time between the MR scan and the biochemical measures was a median of 14 days (range: 0–58).

### Liver

**Fig 1:**
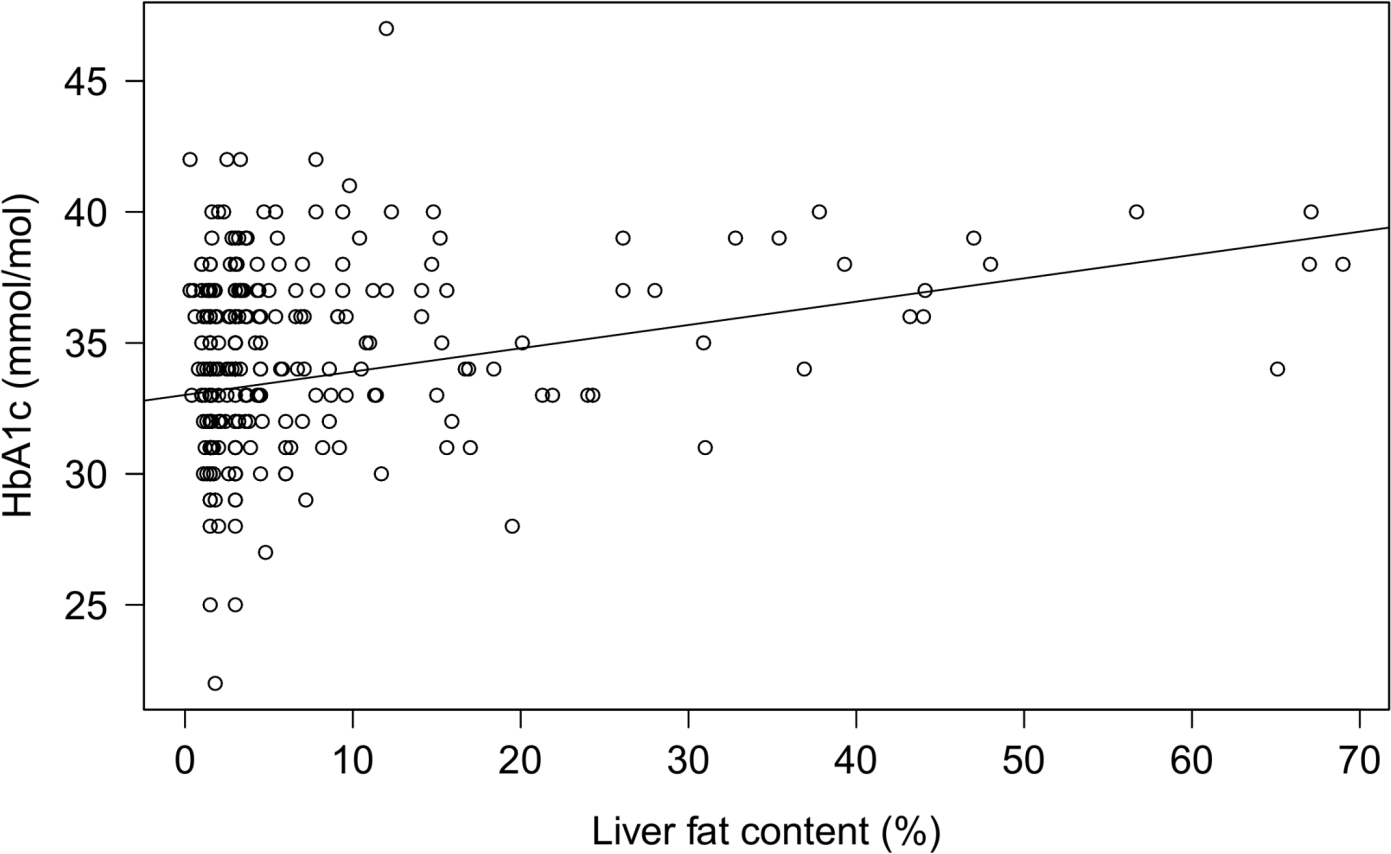
The correlation between LFC and HbA1c. The correlation between proton magnetic resonance spectroscopy measured liver fat content (LFC) and glycosylated hemoglobin (HbA1c) in the 287 overweight/obese children and adolescents: R^2^ = 0.09, *p* = 0.004.

The prevalence of hepatic steatosis among the overweight/obese children and adolescents was 31% (88 of 287), and higher among boys than among girls (41% vs. 23%, *p* = 0.001), whereas only one (3%) of the 40 controls exhibited hepatic steatosis. The sex difference in LFC remained significant after adjusting for BMI SDS (*p* = 0.04), while not in the hepatic steatosis fraction (*p* = 0.24). In the overweight/obese group, a multiple linear regression model adjusted for age, sex, and pubertal development showed that accumulation of fat in the liver associated positively with BMI SDS, VAT, SAT, serum insulin, HbA1c, and HOMA-IR, and inversely with HDL cholesterol (Table 2). BMI SDS, VAT, SAT, and HbA1c remained significantly associated with LFC after adjustment for BMI SDS and MFC (Table 2). The correlation between LFC and HbA1c is shown in Fig 1.

### Muscle

**Fig 2:**
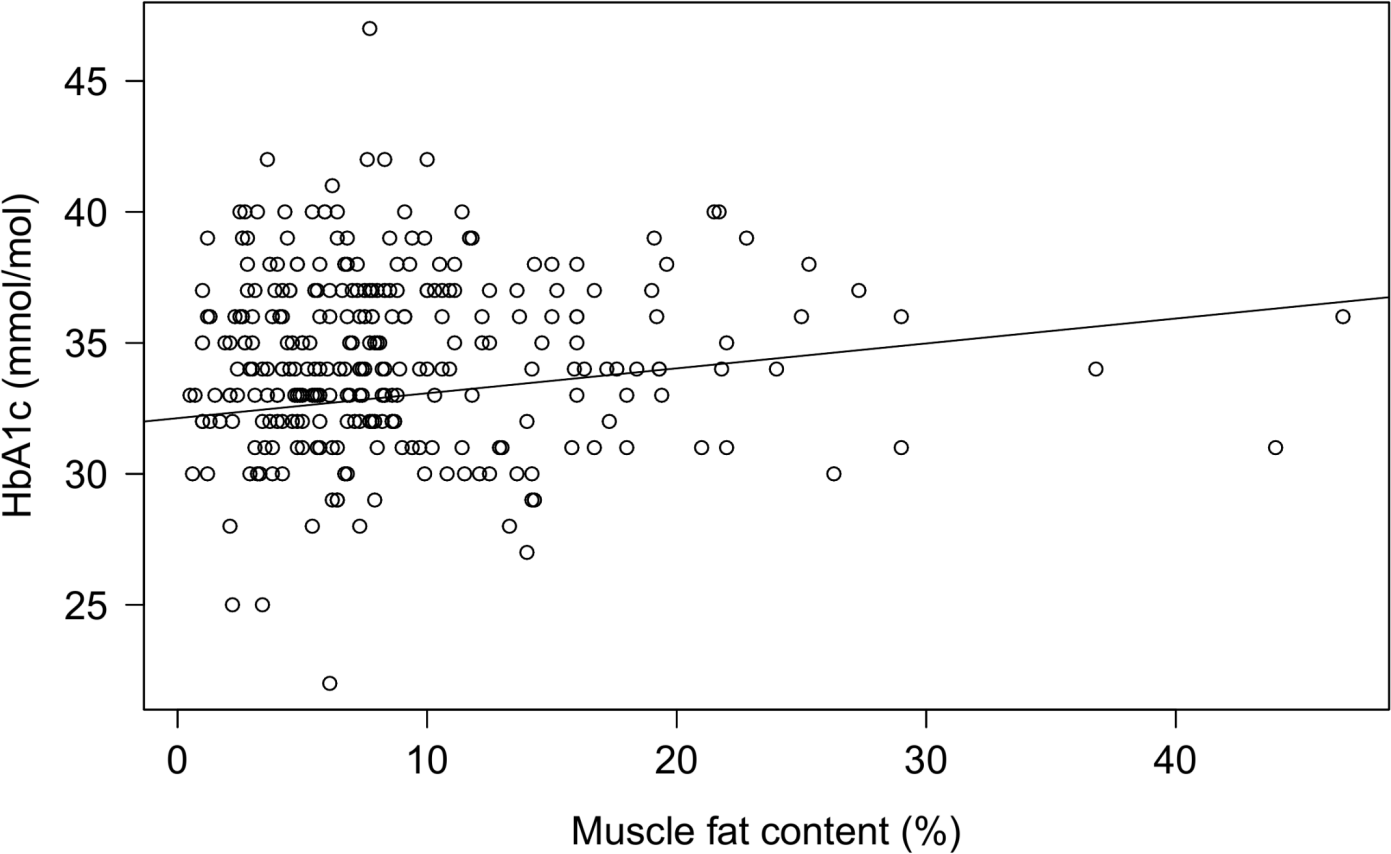
The correlation between MFC and HbA1c. The correlation between proton magnetic resonance spectroscopy measured muscle fat content (MFC) and glycosylated hemoglobin (HbA1c) in the 287 overweight/obese children and adolescents: R^2^ = 0.07, *p* = 0.04.

The prevalence of muscular steatosis among the overweight/obese children and adolescents was 68% (196 of 287) compared with 10% (4 of 40) among the lean controls, with no significant differences between boys and girls (Table 1). In the overweight/obese group, multiple linear regression adjusted for age, sex, and pubertal development showed that overall accumulation of muscle fat associated positively with BMI SDS, VAT, and HbA1c and inversely with HDL cholesterol (Table 3). BMI SDS and HbA1c remained significantly associated with MFC after adjustment for BMI SDS (Table 3). The correlation between MFC and HbA1c is shown in Fig 2.

Further, multiple linear regression in the overweight/obese group showed that the intramyocellular accumulation of fat associated positively with BMI SDS and HbA1c and inversely with HDL cholesterol, which remained significant when adjusting for BMI SDS and LFC (Table 4).

EMCL showed an association with VAT, although this was not present when adjusting for BMI SDS (Table 5).

The correlation coefficient between IMCL and EMCL was R^2^ = 0.16, p = 7.72e-14. The correlation coefficient between IMCL and MFC was R^2^ = 0.44, p<2e-16. The correlation coefficient between EMCL and MFC was R^2^ = 0.91, p<2e-16.

### Dyslipidemia, IFG, and hypertension

When comparing the clinical variables dyslipidemia, IFG, and hypertension among the cases to the presence of steatosis in a multiple logistic regression model adjusted for age, sex, BMI SDS, and pubertal developmental stage, the OR of having dyslipidemia was 4.2 (95%CI: [1.8; 10.2], *p* = 0.0009) when hepatic steatosis was present, while IFG (OR = 1.3, 95%CI: [0.3; 5.1], *p* = 0.67) and hypertension (OR = 0.8, 95%CI: [0.3; 1.9], *p* = 0.55) were not significantly associated to the presence of hepatic steatosis. When muscular steatosis was present, the OR of having IFG was 3.9 (95%CI: [0.9; 26.5], *p* = 0.09), dyslipidemia 1.5 (95%CI: [0.7; 3.2], *p* = 0.26), and hypertension 0.9 (95%CI: [0.4; 2.1], *p* = 0.89). When exhibiting both hepatic and muscular steatosis (n = 66) as compared to those exhibiting neither hepatic nor muscular steatosis (n = 107), the OR of having dyslipidemia was 5.8 (95%CI: [2.0; 18.6], *p* = 0.002), IFG 3.7 (95%CI: [0.6; 31.6], *p* = 0.17), and hypertension 0.7 (95%CI: [0.2; 2.5], *p* = 0.61). The same relationships were present when analyzing the pooled data from the lean and overweight/obese groups (data not shown).

## Discussion

The overweight/obese children and adolescents had higher prevalence rates of hepatic and muscular steatosis and dyslipidemia than the lean controls, while prevalence rates of IFG were comparable between the groups. Hepatic steatosis associated with dyslipidemia.

The prevalence rates of hepatic steatosis in lean and overweight/obese children and adolescents are comparable to rates reported in other papers [7,24–26]. The prevalence rate of muscular steatosis in 159 overweight children and youths is previously reported by our group and support the present findings [8]. Due to differences in methodologies between studies and the lack of general consensus on the exact level defining muscular steatosis, the information on prevalence rates of muscular steatosis is sparse, also in lean children. The lean group, though, had an IMCL content comparable of other studies [3,23,27].

As in the present study, MRS-measured LFC as a quantitative measure has been associated with estimates of glucose and lipid metabolism in studies of 123 [23] and 97 [28] children. In a study by Schwimmer *et al*. [5], 150 children with biopsy-proven hepatic steatosis had significantly impaired glucose and lipid metabolism as compared to 150 age and sex matched controls without hepatic steatosis, whereas in the present study we did not observe a significant association between hepatic steatosis and IFG, despite a comparable sample size. These results suggest that although LFC as a continuous variable is associated with estimates of glucose metabolism, hepatic steatosis may not be as useful in the clinical setting regarding the diagnosis of IFG.

LFC was positively associated with BMI SDS, VAT, and SAT, which are findings in line with previous studies [5,7,23,28]. In addition, Schwimmer *et al*. [5] reported direct associations between hepatic steatosis and BP in 300 children and adolescents; a relationship that was not observed in the present study despite comparable age and number of study participants.

The presence of muscular steatosis did not associate significantly with neither the diagnoses of dyslipidemia, IFG, nor hypertension, although we observed a tendency of a link between muscular steatosis and IFG. In comparable studies, evidence on the associations between muscular steatosis and obesity-related dyslipidemia, IFG, and hypertension is sparse. On the continuous variables though, MFC has been positively associated with BMI SDS, VAT, and IR [4,8,28], and MRS-measured IMCL was found to associate positively with IR measured by a euglycemic clamp in two studies of 23 lean adults [3] and 28 obese children [13], to IR estimated by an oral glucose tolerance test in 21 obese adolescents [29], and to IR estimated by the triglyceride/HDL-cholesterol ratio in a study sample of 441 pre-pubertal and early pubertal children [30]. No association between IMCL and IR was observed by Bennett *et al*. [23] in a study of 123 pre-pubertal children nor in the present study, where HOMA-IR was used as an estimate of IR. A possible explanation of the lack of association between either fragment of muscle fat and the continuously analyzed variable HOMA-IR in the current study could be found in the diversity of insulin resistance between sexes and stages of pubertal developmental [31] and that HOMA-IR is not an optimal estimate of IR in skeletal muscle [32]. Furthermore, it is possible that the relationship between MFC and IR is mediated by different factors involving fat metabolism in the muscle cell, which result in high levels of muscle fat that are not necessarily associated to IR as observed in ‘the athlete’s paradox’ [33].

As in the present study, Brumbaugh *et al*. [30] showed IMCL to positively associate with BMI. In contrast to our study, Brumbaugh *et al*. [30] did not find any associations between IMCL and HDL-cholesterol, but instead between IMCL and VAT and triglyceride levels [30]. The aforementioned studies by Brumbaugh *et al*. [30] and Larson-Meyer *et al*. [28] demonstrated a direct association between IMCL and systolic BP, which was not observed in the present study.

Associations between EMCL and the investigated variables of glucose and lipid metabolism, fat distribution, and BP exhibit low coverage in the literature. In a pediatric study, Sinha *et al*. (n = 22) [27] reported a direct association between EMCL and euglycemic clamp measured IR, but not lipid metabolism. In contrast, Weiss *et al*. (n = 28) [13] found no association between EMCL and euglycemic clamp measured IR. We observed a direct association between EMCL and VAT; an association that did not persist after adjusting for BMI SDS, suggesting that the EMCL may be regulated according to the degree of obesity.

### IFG

In two nationwide cohorts of obese children and adolescents [34], prevalence rates of impaired fasting glucose were 6% in Germany (n = 32,907) and 17% in Sweden (n = 2,726) of which the prevalence rates in the present study falls right in between. Among 777 adolescents in an NHANES study, 13% exhibited impaired fasting glucose and the prevalence rates were 10% among the normal weight, 15% for those with a BMI in the 85^th^ to 95^th^ percentile range, and 23% for those with a BMI above the 95^th^ percentile [35]. In the present study, comparable prevalence rates of IFG were observed between cases and controls, despite significant differences in ectopic fat accumulation. This could be explained by the controls being older and more advanced in pubertal development and thus most likely more influenced by the physiological insulin resistance that may develop during puberty and which tends to worsen their glucose metabolism during the course of sexual development [36].

### Sex differences

Among the overweight/obese, the levels of BMI SDS and LFC, as well as the fraction of hepatic steatosis, were higher in boys than in girls, in accordance with previous papers [7,14,24]. The augmented accumulation of fat in the liver among boys may be due to a less extensive capacity to store fat in the adipose tissue, as compared to girls; a difference that might partly be influenced by sex steroid and glucocorticoid concentrations [37]. Moro *et al*. observed, among 48 adults, that women had a significantly higher IMCL than their male peers [38], whereas no sex difference in IMCL was observed in the present study.

### Strengths and limitations

Among the strengths of the present study is that, instead of the absolute values, SDS was used for the evaluation of BP and BMI, which takes growth and development during childhood and adolescence into account. Another strength is the relatively large number of participants with liver and muscle fat assessed by MRS and related to concomitant measures of pubertal developmental stage, blood pressure, anthropometrics, and fasting insulin, glucose, and lipids. A strength of the study is the objectively measured pubertal staging of the cases, while a limitation of the study is the self-reported staging of pubertal developmental among the controls.

Among other limitations of this study is the relatively small number of controls, as compared to the number of cases. The analyses of associations were made without adjusting for multiple comparisons, which increases the chance of type I errors. Furthermore, all measures of anthropometry and biochemistry were not assessed on the same day as the MR scan, hereby allowing natural day-to-day biological variations to affect the results. Another limitation is that IR was measured as a proxy by HOMA-IR and not by the glucose clamp technique, which is the gold standard [39], although HOMA-IR is a relatively accepted surrogate estimate of IR [40]. The lack of physical activity, fitness, or nutrition measures limits the clinical utility of the present findings.

## Conclusion

Ectopic fat deposition in liver and muscle tissue is prevalent in childhood obesity. The observed links between hepatic steatosis and dyslipidemia, and ectopic fat and obesity-related metabolic dysfunctions—particularly glycosylated hemoglobin, suggest a biologically important increased cardiovascular disease risk.

## Supporting Information

S1 Table Baseline characteristics of 287 overweight/obese (cases) and 40 lean (controls) children and adolescents. Just like Table 1, with data presented as means ± standard deviation (instead of medians and interquartile range). BMI, body mass index; diaBP, diastolic blood pressure; EMCL, extramyocellular lipid content; HbA1c, glycosylated hemoglobin; HDL, high density lipoprotein; HOMA-IR, homeostatic model assessment of insulin resistance; IMCL, intramyocellular lipid content; LDL, low density lipoprotein; LFC, liver fat content; MFC, muscle fat content; SAT, subcutaneous adipose tissue volume; SDS, standard deviation score; sysBP, systolic blood pressure; VAT, visceral adipose tissue volume.
(DOCX)
