# Supplementary material for: 1H-MRS Measured Ectopic Fat in Liver and Muscle in Danish Lean and Obese Children and Adolescents
Source: PLoS One. 2015 Aug 7;10(8):e0135018. doi: 10.1371/journal.pone.0135018 (PMC4529156; doi:10.1371/journal.pone.0135018)
Supplement: S1 Table — Just like Table 1, with data presented as means ± standard deviation (instead of medians and interquartile range). BMI, body mass index; diaBP, diastolic blood pressure; EMCL, extramyocellular lipid content; HbA1c, glycosylated hemoglobin; HDL, high density lipoprotein; HOMA-IR, homeostatic model assessment of insulin resistance; IMCL, intramyocellular lipid content; LDL, low density lipoprotein; LFC, liver fat content; MFC, muscle fat content; SAT, subcutaneous adipose tissue volume; SDS, standard deviation score; sysBP, systolic blood pressure; VAT, visceral adipose tissue volume. (DOCX) [file pone.0135018.s001.docx]

|  | **Cases**  **Girls ♀** | **Controls Girls ♀** | ***p*** | **Cases**  **Boys ♂** | **Controls Boys ♂** | ***p*** | **Cases**  ♀ *vs* ♂ | **Controls** ♀ *vs* ♂ |
| --- | --- | --- | --- | --- | --- | --- | --- | --- |
| N | 165 | 19 |  | 122 | 21 |  | 287 | 40 |
| Age, *years* | 13.4 ± 2.4 | 14.3 ± 2.5 | *0.13* | 12.9 ± 2.0 | 13.9 ± 3.0 | *0.08* | *0.07* | *0.77* |
| BMI SDS | 2.71 ± 0.53 | -0.02 ± 0.62 | ***<0.0001*** | 3.08 ± 0.60 | 0.11 ± 0.63 | ***<0.0001*** | ***<0.0001*** | *0.54* |
| VAT, *cm^3^* | 84 ± 45 | 18 ± 10 | ***<0.0001*** | 94 ± 50 | 19 ± 14 | ***<0.0001*** | ***0.04*** | *1.00* |
| SAT, *cm^3^* | 316 ± 118 | 65 ± 31 | ***<0.0001*** | 308 ± 110 | 45 ± 24 | ***<0.0001*** | *0.71* | ***0.02*** |
| LFC, % | 5.2 ± 7.2 | 2.0 ± 1.3 | ***0.002*** | 10.3 ± 15.1 | 1.7 ± 0.6 | ***<0.0001*** | ***0.0005*** | *0.46* |
| Hepatic steatosis, *fraction* | 23% | 5% | *0.08* | 41% | 0% | ***<0.0001*** | ***0.001*** | *0.48* |
| MFC, % | 8.6 ± 6.7 | 2.7 ± 2.1 | ***<0.0001*** | 9.1 ± 6.7 | 2.2 ± 2.2 | ***<0.0001*** | *0.72* | *0.51* |
| Muscle steatosis, *fraction* | 68% | 11% | ***<0.0001*** | 68% | 10% | ***<0.0001*** | *1.00* | *1.00* |
| IMCL, % | 2.3 ± 2.3 | 1.0 ± 1.0 | ***0.0003*** | 2.5 ± 2.2 | 0.8 ± 0.8 | ***<0.0001*** | *0.14* | *0.94* |
| EMCL, % | 6.5 ± 5.6 | 0.6 ± 1.5 | ***<0.0001*** | 6.7 ± 5.4 | 1.3 ± 1.7 | ***<0.0001*** | *0.91* | *0.44* |
| Triglyceride, *mmol/l* | 1.2 ± 0.6 | 0.8 ± 0.3 | ***0.0002*** | 1.2 ± 0.8 | 0.6 ± 0.2 | ***<0.0001*** | *0.08* | *0.06* |
| HDL cholesterol, *mmol/l* | 1.2 ± 0.3 | 1.6 ± 0.3 | ***<0.0001*** | 1.2 ± 0.3 | 1.7 ± 0.5 | ***<0.0001*** | *0.10* | *0.22* |
| LDL cholesterol, *mmol/l* | 2.5 ± 0.8 | 2.4 ± 0.6 | *0.82* | 2.6 ± 0.8 | 2.1 ± 0.7 | ***0.005*** | *0.18* | *0.07* |
| Non-HDL cholesterol, *mmol/l* | 3.0 ± 0.8 | 2.8 ± 0.6 | *0.10* | 3.1 ± 0.9 | 2.8 ± 0.7 | ***0.0003*** | *0.56* | ***0.03*** |
| Plasma glucose, *mmol/l* | 5.0 ± 0.5 | 5.1 ± 0.4 | *0.28* | 5.2 ± 0.4 | 5.0 ± 0.5 | *0.34* | ***0.001*** | *0.67* |
| Serum insulin, *pmol/l* | 116 ± 114 | 75 ± 32 | *0.05* | 115 ± 122 | 48 ± 22 | ***<0.0001*** | *0.69* | ***0.004*** |
| HbA1c, *mmol/l* | 34 ± 3 | 36 ± 2 | ***0.03*** | 35 ± 3 | 34 ± 3 | *0.45* | *0.73* | ***0.02*** |
| HOMA-IR | 3.82 ± 3.88 | 2.48 ± 1.12 | *0.09* | 3.85 ± 4.32 | 1.57 ± 0.76 | ***<0.0001*** | *0.43* | ***0.007*** |
| sysBP SDS | 2.24 ± 1.15 | 1.48 ± 0.95 | ***0.001*** | 2.11 ± 1.34 | 2.38 ± 1.41 | *0.44* | *0.27* | ***0.047*** |
| diaBP SDS | 1.06 ± 0.78 | 0.47 ± 0.60 | ***0.001*** | 0.70 ± 0.64 | 0.38 ± 0.65 | *0.12* | ***0.0004*** | *0.98* |
| Tanner stage | 3 ± 1 | 3 ± 2 | *0.79* | 2 ± 1 | 3 ± 2 | *0.08* | ***<0.0001*** | *0.65* |
